# The potential role of MR based radiomic biomarkers in the characterization of focal testicular lesions

**DOI:** 10.1038/s41598-021-83023-4

**Published:** 2021-02-10

**Authors:** Giacomo Feliciani, Lorenzo Mellini, Aldo Carnevale, Anna Sarnelli, Enrico Menghi, Filippo Piccinini, Emanuela Scarpi, Emiliano Loi, Roberto Galeotti, Melchiore Giganti, Gian Carlo Parenti

**Affiliations:** 1 Istituto Scientifico Romagnolo per lo Studio e la Cura dei Tumori (IRST) IRCCS, Via P. Maroncelli 40, 47014 Meldola, FC Italy; 2 Department of Morphology Surgery and Experimental Medicine, University of Ferrara, Via L. Ariosto 34, 44121 Ferrara, Italy; 3 Department of Radiology, University Hospital of Ferrara, Via A. Moro 8, 44124 Ferrara, Italy

**Keywords:** Cancer imaging, Testicular cancer

## Abstract

How to differentiate with MRI-based techniques testicular germ (TGCTs) and testicular non-germ cell tumors (TNGCTs) is still under debate and Radiomics may be the turning key. Our purpose is to investigate the performance of MRI-based Radiomics signatures for the preoperative prediction of testicular neoplasm histology. The aim is twofold: (i), differentiating TGCTs and TNGCTs status and (ii) differentiating seminomas (SGCTs) from non-seminomatous (NSGCTs). Forty-two patients with pathology-proven testicular neoplasms and referred for pre-treatment MRI, were retrospectively enrolled. Thirty-two out of 44 lesions were TGCTs. Twelve out of 44 were TNGCTs or other histologies. Two radiologists segmented the volume of interest on T2-weighted images. Approximately 500 imaging features were extracted. Least Absolute Shrinkage and Selection Operator (LASSO) was applied as method for variable selection. A linear model and a linear support vector machine (SVM) were trained with selected features to assess discrimination scores for the two endpoints. LASSO identified 3 features that were employed to build fivefold validated linear discriminant and linear SVM classifiers for the TGCT-TNGCT endpoint giving an overall accuracy of 89%. Four features were employed to build another SVM for the SGCT-SNGCT endpoint with an overall accuracy of 86%. The data obtained proved that T2-weighted-based Radiomics is a promising tool in the diagnostic workup of testicular neoplasms by discriminating germ cell from non-gem cell tumors, and seminomas from non-seminomas.

## Introduction

Over the last decades, there has been a steady worldwide increase in the incidence of testicular cancer^1^. The majority of these tumors are the testicular germ cell tumors (TGCTs), which are then divided into two broad classes: seminomatous (SGCTs) and non-seminomatous germ cell tumors (NSGCTs). On this categorisation depend both the treatment and the prognosis^2^. For instance, SGCT is more sensitive to radio and chemotherapy and thus has a better prognosis. Although ultrasonography (US), including conventional grey-scale and color-doppler, still maintains the primary role in the diagnostic workup of scrotal pathology, magnetic resonance imaging (MRI)^3^ has emerged as a supplemental imaging modality, which is mainly recommended as a problem-solving tool in challenging cases^4^. Hence, MRI may provide additional information and help to clarify inconclusive or equivocal US findings in order to reduce the incidence of unnecessary surgery^4,5^. Albeit MRI may facilitate the differentiation between benign and malignant tumors^6^, imaging alone is sometimes insufficient in making a clear distinction among testicular lesions. Previous studies have underlined the role of qualitative radiological assessment based on T1- and T2-weighted MR images that help to differentiate between seminomas and non-seminomatous tumors^7^. These studies have been further supported by quantitative investigation on diffusion weighted imaging (DWI) which have reported similar accuracy in discriminating SGCT–NSGCT statu^8,9^; however, current existing data do not unequivocally support the role of DWI in being able to differentiate TGCT from testicular non-germ cell tumors (TNGCT)^10^. Given the rarity of these tumors, these results were obtained from small cohorts and still require validation. However, in the past decade, the breakthroughs in artificial intelligence and high-throughput computing have accelerated the application of radiomic analysis to medical imaging with the aim of guiding clinical decision-making.

The drive behind the spread of Radiomics is the attempt to derive quantitative features from digital images in order to provide information which is not obvious to human interpretation alone^11,12^. Radiomics appears to supply diverse imaging biomarkers in different medical fields, although medical oncology represents the main area of research, since such image analysis may be of help in tumor detection, diagnosis, prognostication and prediction of response to treatment^11,13^. Indeed, in a recent publication, Zhang et al*.*^14^, developed a radiomic signature to quantitatively discriminate seminomas from non-seminomatous tumors obtaining higher classification rate compared to the other standard MRI-based techniques (e.g. visual inspection, ADC and DWI value).

Therefore, this study extends and improves the work of Zhang et al*.* by investigating the diagnostic performance of internally validated radiomic models in characterizing testicular neoplasms and more specifically differentiating between TGCTs and TNGCTs where classification is still under debate. Our findings show that in this field, MRI and Radiomics together allow accurate characterization of testicular lesions, successfully guiding clinical decision-making.

## Methods

### Patient selection

**Figure 1:**
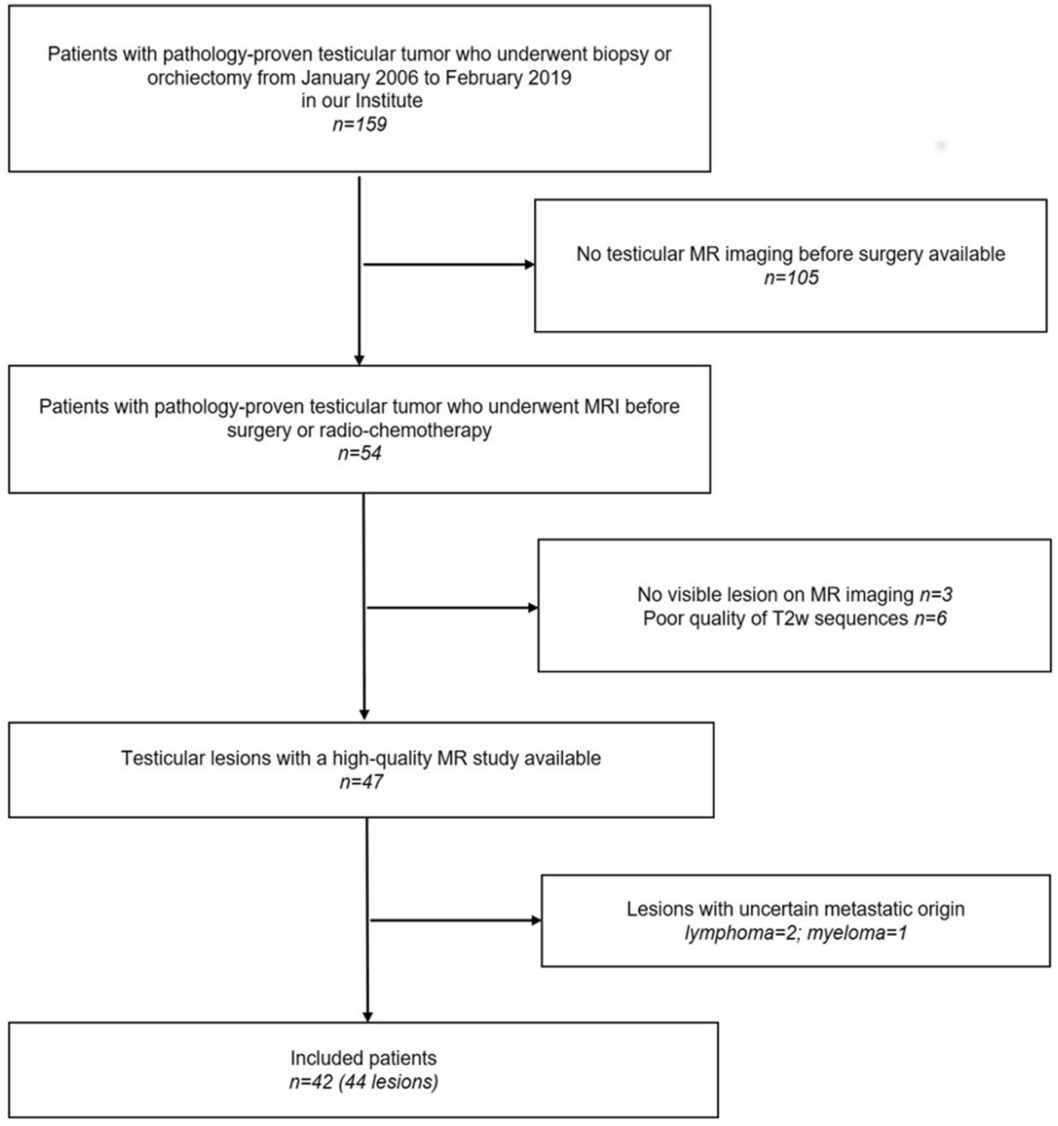
Flowchart summarizing patient accrual.

In this observational retrospective study, approved by the institutional review board of the *Azienda USL della Romagna* (informed consent is published in integral part on the website of *Azienda USL della Romagna prot. N. 1683*), a dataset of MR images of 42 patients who were referred for pathology has been analyzed. All research steps were performed in accordance with relevant regulations and informed consent was waived by the IRB. After biopsy or orchiectomy, the Pathology Department of *Azienda USL della Romagna* provided us with confirmation of histological diagnosis, in all testicular tumors which had undergone surgery from January 2006 to February 2019. All patients who had a scrotal MRI available in our imaging archive system (Carestream VuePACS, Carestream Health, Rochester, NY, USA) were consequently selected. Exclusion criteria were the following: (a) patients who underwent MRI after surgical or radiotherapy and/or chemotherapy treatment; (b) poor quality of the MR images due to movement artefacts; (c) no visible lesion on MRI; (d) not primary testicular tumor (Fig. 1). MRI was performed in clinical practice as a second-level problem-solving tool when sonographic findings were equivocal or inconclusive, or following a request from the urology department to obtain a detailed local staging of a testis mass previously identified with US. The patient cohort was aged from 7 to 79 years old (average 39.3 ± 14.3 years). One of the patients had a bilateral classic seminoma and one had two different neoplasms years apart. We excluded 2 patients with testicular lymphoma and 1 with testicular localization of myeloma because of the uncertain metastatic origin; 1 more patient with classic seminoma was discarded due to poor image quality, so the final dataset consisted of 42 patients. Therefore, we analyzed MR studies of 44 testicular lesions (patient and lesion features are summarized in Table 1). Time difference between MRI and histologic final diagnosis was 25 ± 15 days. Thirty-two out of 44 were histologically classified as TGCTs, including 23 classic seminomas and 9 NSGCTs (7 mixed germ cell tumors and 2 embryonal cell carcinomas). Twelve lesions out of 44 were TNGCTs or other histological types: 7 Leydig cell tumors, 2 Sertoli cells tumors, 2 adenomatoid tumors and 1 epidermoid tumor. For each lesion, laterality (left/right) and size have been considered; germ cell tumors were staged according to the *8th Edition of the American Joint Committee on Cancer (AJCC) Staging Manual*. For a more detailed description of the lesions, also several visual features were analyzed in Supplementary Table S1, created by following indications found in^7^. These features included signal intensity of the lesion compared to normal parenchyma, presence of necrotic or haemorrhagic areas, presence of tumor capsule. Furthermore, bandlike structures on T2w images were considered fibrovascular septa and the contrast of these septa was also analyzed.

**Table 1:** Patient demographics and lesion features.

Germ cell tumors		
AGE (year)	Average ± standard deviation	36.8 ± 9
LATERALITY	Right/left	19/13
SIZE (maximum diameter—cm)	Average ± standard deviation	3.2 ± 2.4
STAGING (T)	pT1/pT2/pT3/pT4	17/13/2/0

Non germ cell tumors		
AGE (year)	Average ± standard deviation	39.1 ± 18.6
LATERALITY	Right/left	4/8
SIZE (maximum diameter—cm)	Average ± standard deviation	0.94 ± 0.46

### MR imaging protocol and radiomic analysis

**Figure 2:**
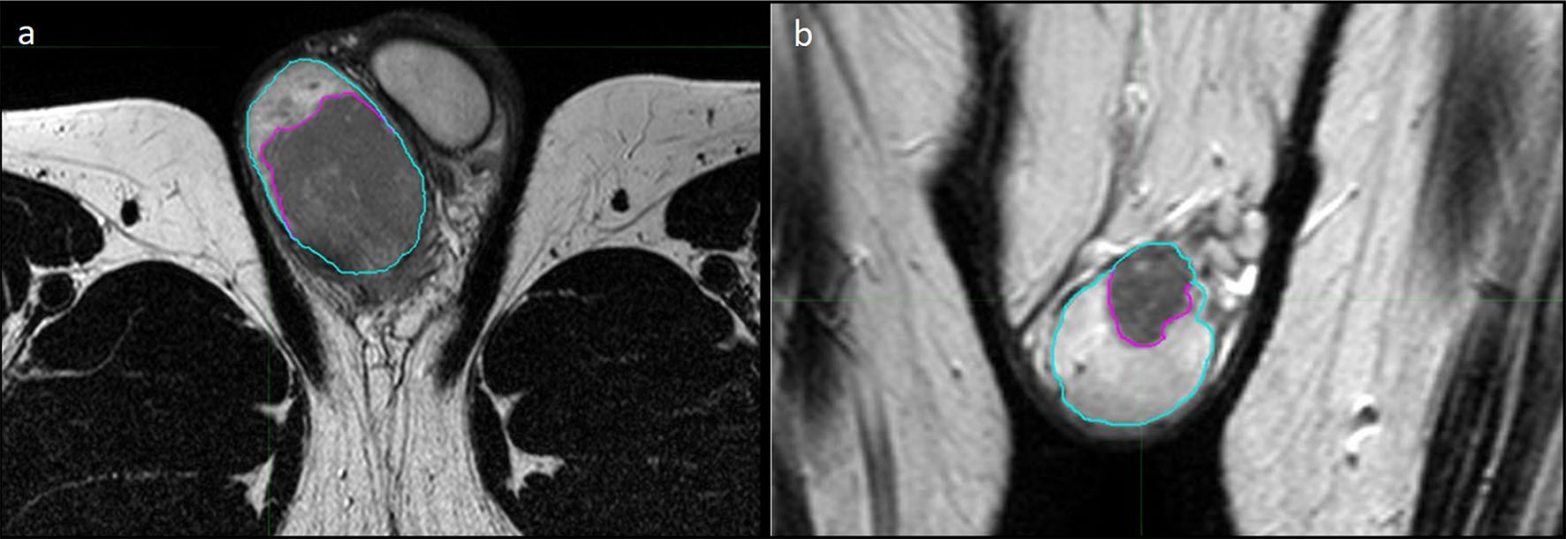
MR images showing the segmentation process in a 27-year-old man with testicular seminoma (**a**) and a 31-year-old man with Leydig cell tumor (**b**), axial and coronal T2-weighted images, respectively. Testicles are contoured in blue, whereas neoplasms are contoured in violet.

MR studies were acquired using a 1.5 T MR Scanner (Achieva Philips, Philips Healthcare, Best, Netherlands) by using a surface coil (Philips Sense Flex Medium coil). All the patients were placed in the scanner in the supine position, feet first. After adequate support and positioning of the scrotum, elevated by placing a towel between the thighs with the penis raised and fixed to the lower abdominal wall, the surface coil was placed over a second towel covering the scrotum. Peripheral venous access (19-gauge) was obtained in an antecubital fossa vein. All the MRI study protocols included T1-weighted (T1w) sequences before and after paramagnetic contrast agent administration and T2w sequences in the axial, coronal and sagittal plane; some of the examinations also included DWI sequences and derived apparent diffusion coefficient (ADC) maps. T2w sequences were selected for radiomic analysis since they are the most complete imaging set for each patient and are the best for lesion detection, localization and characterization, providing essential information on neoplastic tissue and anatomic detail^4^. MRI parameters for T2w sequence are summarized in Table 2. Spatial resolution varied from 0.3 to 0.7 mm in the axial direction and from 3 to 4 mm in the *z*-direction. Resampling of the images was performed before contouring and radiomic analysis in order to uniform dataset to an average resolution of 0.5/0.5/3.5 mm. Contouring of the patient lesions was performed on the T2w sequences (Fig. 2a,b) through consensus between two expert radiologists. First, second and higher order features were extracted with the open source *MATLAB* (The MathWorks, Inc., MA, USA) based software *Standardized Environment for Radiomics Analysis* (SERA) version 2.1^15^ that is an *Image Biomarker Standardisation Initiative* (IBSI) compliant tool^16^. A quick guide on setting up and run SERA for feature extraction is present in the readme file of SERA available at: https://github.com/ashrafinia/SERA. First order features were derived from the histogram of voxel intensities. Second and higher order features were calculated from Intensity size-zone, co-occurrence and run-length based matrices. A detailed description of the software and of the 487 imaging features extracted can be found in^15^ and in^16^, respectively. Grey level quantization was fixed to 64 bins between the minimum and maximum value inside the region of interest (ROI).

**Table 2:** Turbo Spin-Echo T2-weighted image acquisition parameters.

Acquisition parameter	Value
Slice thickness	3.5 mm 3–4 mm
Min. slice gap	0
Repetition time	5899 ms
Echo time	120 ms
Flip angle	90°

Field of view	
Right/left dimension	160 mm
Anterior/posterior dimension	90 mm
Foot/head dimension	160 mm

### Statistical analysis

The endpoint of this study was to investigate the diagnostic performance of textural features against two different biopsy responses. The first response was to discriminate between germinal and non-germinal lesions, whereas the second was to assess whether the tumor was a seminoma or not. Radiomics feature extracted with SERA were statistically analysed with *R* and the open source software *RStudio*^17^ to assess their discrimination power between germinal (TGCTs labelled as 0) and non-germinal (TNGCTs labelled as 1). *Least Absolute Shrinkage and Selection Operator* (LASSO) with an integrated fivefold cross validation algorithm was applied as the method for variable selection. LASSO identifies the most significant features that are associated with our endpoints. The same procedure for significant features identification was performed again for the discrimination of SGCTs (labelled as 0) versus NSGCTs (labelled as 1). LASSO was applied 100 times to account for its iterative nature. The features appearing more frequently in the results of LASSO were selected to build the final predictive model, considering a maximum of 4 features to avoid overfitting given the small size of the dataset. A correlation test was performed among significant features to remove residual redundancy through Spearman-Rho correlation coefficient. Features which correlated with each other more than ρ = 0.5 were discarded. *MATLAB R2018a statistical toolbox*^18^ was employed to generate a validated classifier and evaluate its performance. All the designed scripts are provided on request. In order to reduce overfitting of the classifiers, fivefold cross validation has been performed. A linear model and a linear support vector machine (SVM) were trained to assess discrimination scores of statistical models. Confusion matrices were employed to visualize the classifiers’ performances. Due to the class imbalances of the dataset, precision, recall and F1-score were calculated to evaluate the classifiers’ efficiency.

### Ethics approval and consent to participate

Written informed consent was waived by the Institutional Review Board.

## Results

**Figure 3:**
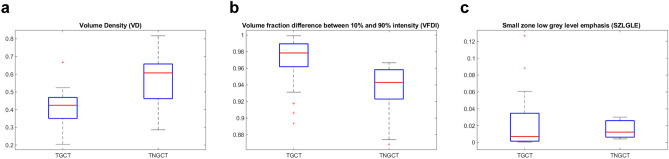
In (**a**) we show VD calculated values for TGCT and TNGCT tumor cell cancers. The same applies for VFDI in (**b**) and in **C** for SZLGLE.

**Figure 4:**
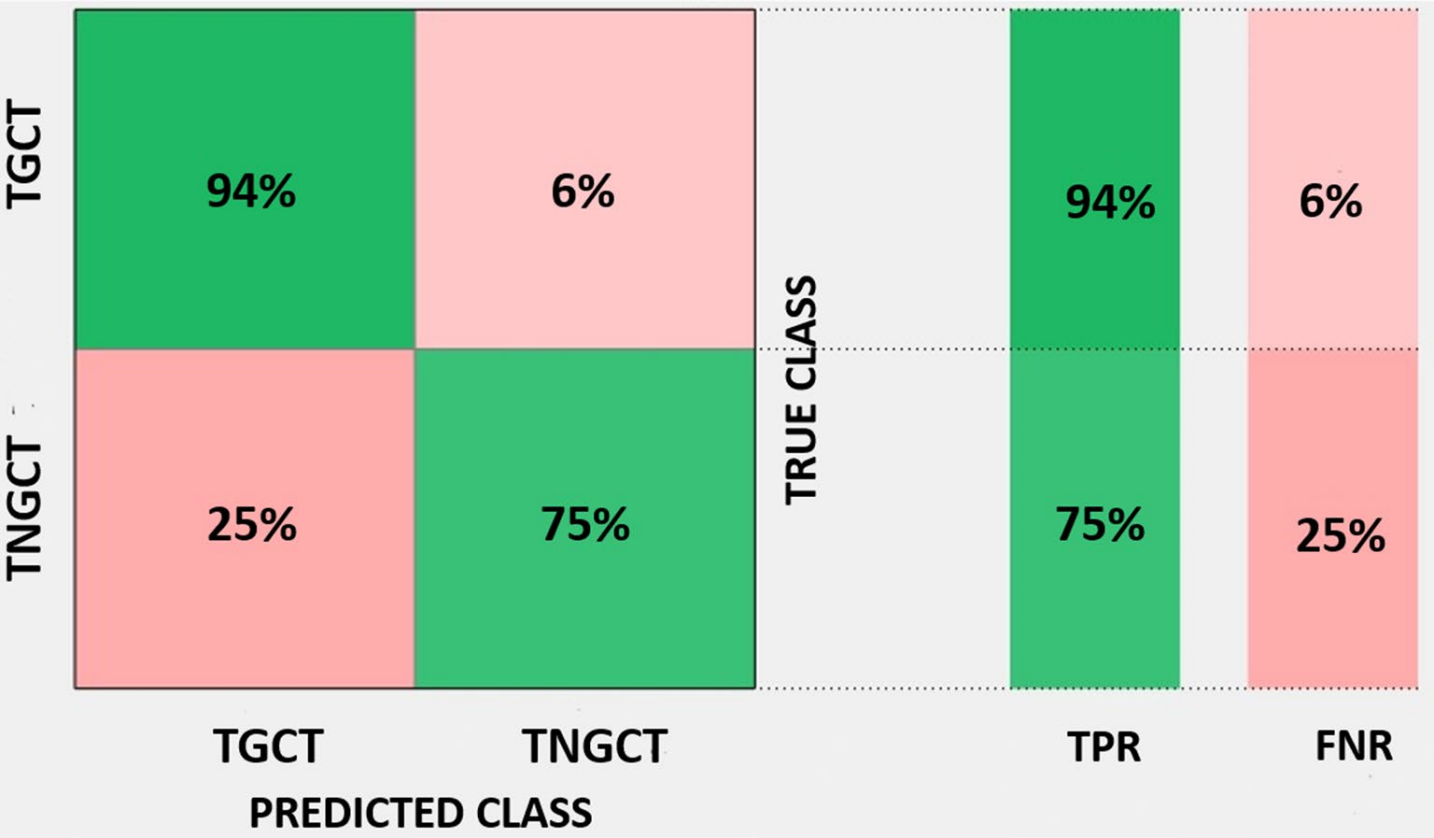
Confusion matrix of the fivefold cross-validated linear SVM trained to discriminate TGCT from TNGCT status with an accuracy of 89% and a TPR of 95% and 75% for TGCT and TNGCT respectively.

**Figure 5:**
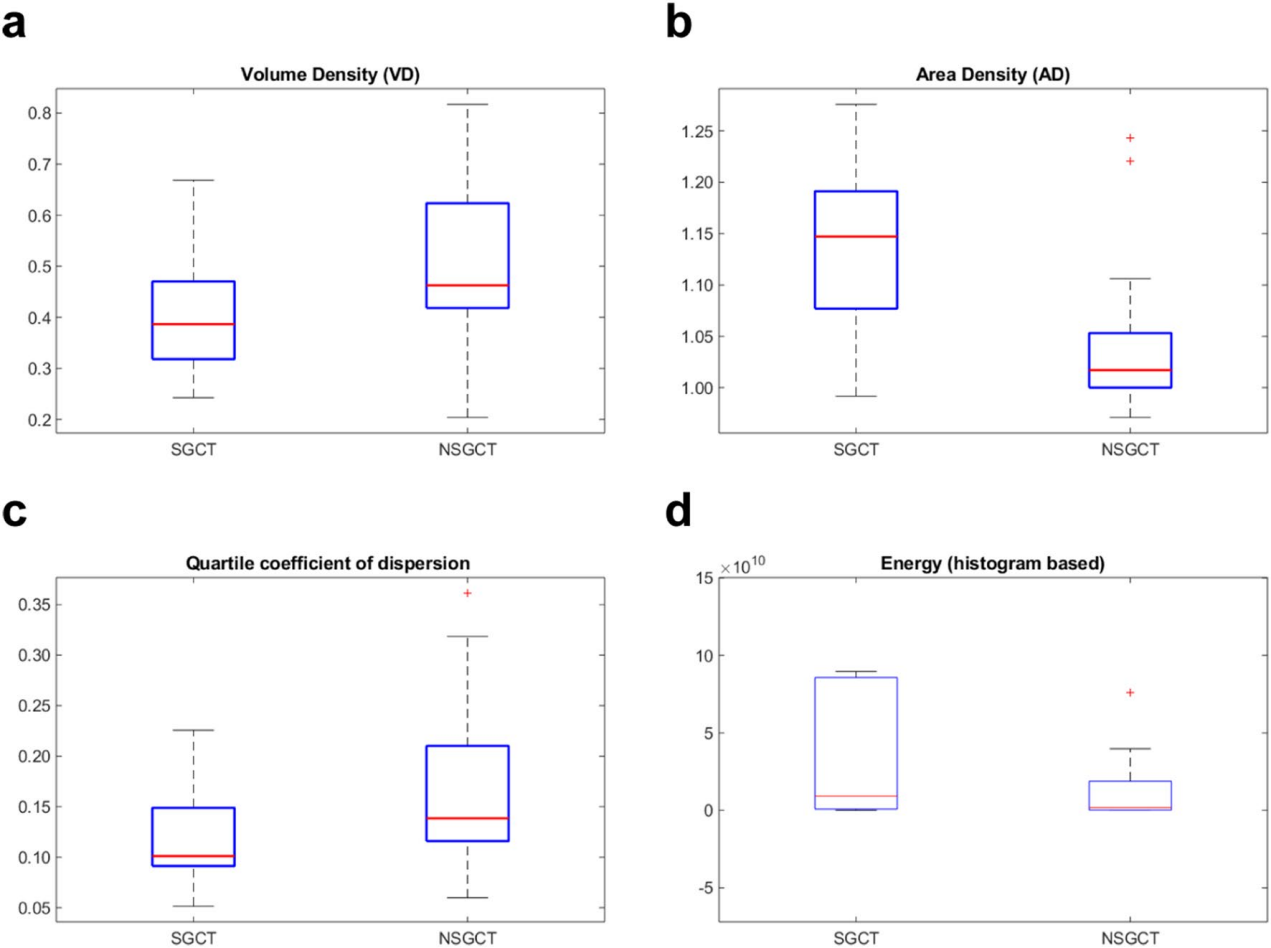
In (**a)** we show VD calculated values for SGCT and NSGCT tumor cell cancers. The same applies for AD, Quartile coefficient of dispersion and energy in (**b**), (**c**) and (**d**) respectively.

**Figure 6:**
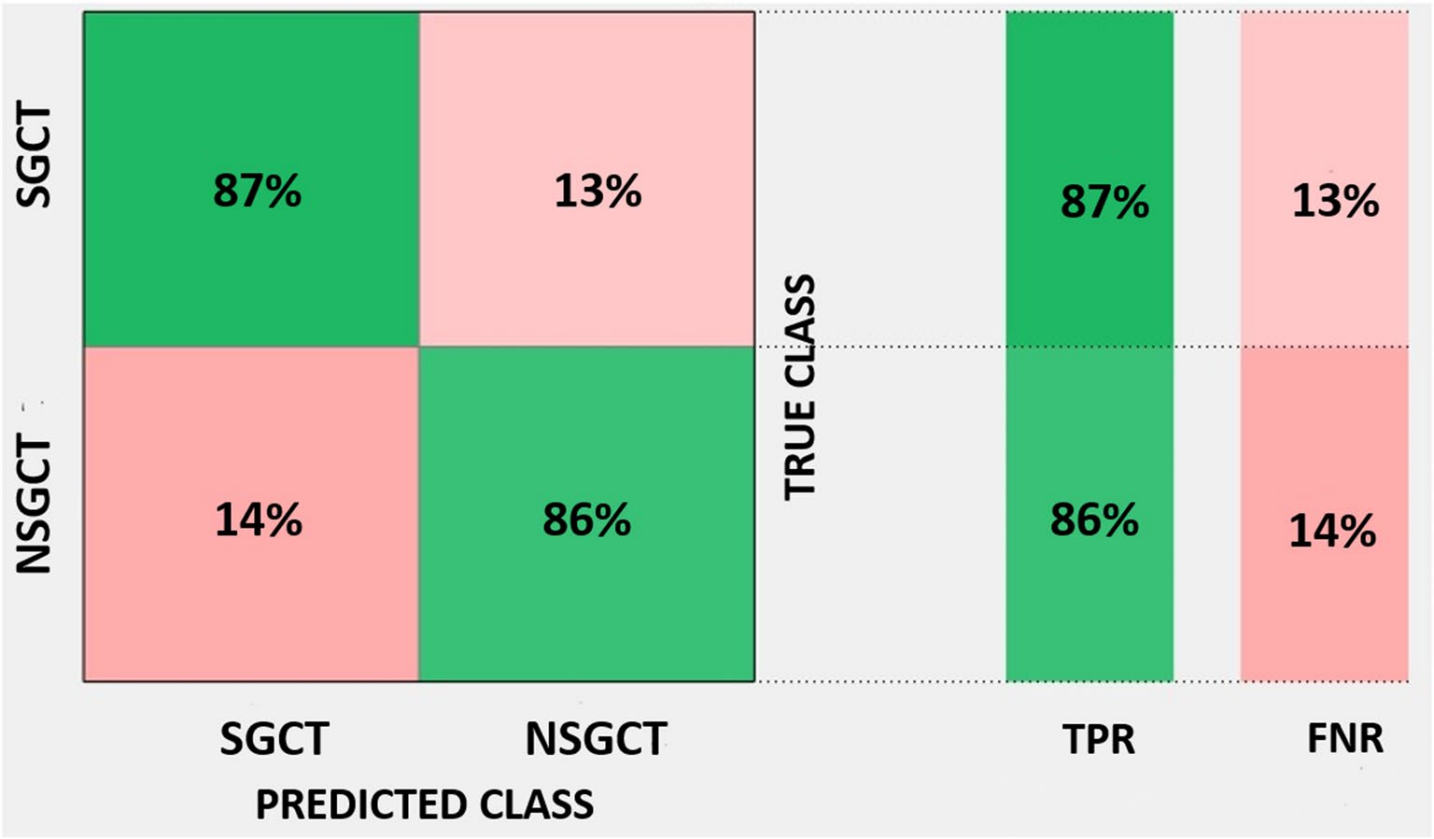
Confusion matrix of the fivefold cross-validated linear SVM trained to discriminate SGCT from NSGCT status with an accuracy of 86% and a TPR of 87% and 86% for SGCT and SNGCT respectively.

From the 44 lesions finally identified, a total of 487 features were extracted. LASSO algorithm was independently applied for the two endpoints of this study. In the pool of features identified by LASSO and after evaluating the correlations with spearman ρ we finally identified 3 features for the association with TGCT-TNGCT discrimination endpoint and 4 features for the SGCT-NSGCT status. Volume density (VD), Volume fraction difference between 10 and 90% intensity (VFDI) and small zone low grey level emphasis (SZLGLE) were employed to build fivefold validated linear discriminant and linear SVM classifiers for the TGCT-TNGCT endpoint. For a detailed description of the features we remind to Supplementary Material S3 taken from the IBSI manual in^16^. In Fig. 3 are represented the boxplots of the three features selected to train the classifiers. The strongest classifier resulted to be the SVM with an overall accuracy of 89%. Figure 4 details the classification performances of the model which has a true positive rate (TPR) of 94% and 75% in predicting TGCT and TNGCT, respectively. The precision of the model is 0.91 for TGCT prediction and 0.81 for NTGCT which is the minority class in the dataset. The recall is 0.93 for TGCT and 0.75 for NTGCT. While, the F1-score is 0.92 for TGCT and 0.78 for NTGCT. VD, Area density (AD), Quartile coefficient of dispersion and energy were identified by LASSO to discriminate the SGCT-NSGCT status. In Fig. 5 we show boxplots of the 4 features selected for classifier training. The best classifier turned out to be a linear SVM with an overall accuracy of 86% and a TPR of 86% and 87% in predicting SGCT and NSGCT, respectively, as reported in Fig. 6. The precision of the model is 0.84 for SGCT and 0.89 for NSGCT which is in this model the minority class. The recall is 0.91 and 0.81, respectively. The F1-score of the model is 0.87 for SGCT and 0.85 for NSGCT.

## Discussion

This study evaluated the ability of T2w MR-based quantitative analysis to help differentiate TGCT from TNGCT tumors and SGCT from SNGCT. In the United States, testicular cancer represents the most common malignancy among men aged 15–44, with almost 9600 new cases estimated in 2019^19^; in young men, germ cell-derived tumors constitute by far the vast majority of testis neoplasms (almost 95%), with benign sex cord–stromal tumors representing approximately the remaining 5%^20^. Moreover, germ cell tumors are almost equally composed of seminomas and non-seminomas^21^, with differences in treatment strategies and prognosis^22^. Advances in multimodality treatments, including surgery, chemotherapy and radiation, have yielded a noticeable decline in mortality rates of testis cancer, particularly when the diagnosis is made early in the clinical course; The preoperative diagnosis with US has been shown to have a 92–98% sensitivity and a 95–99,8% specificity^21^ but cannot be used to accurately predict tumor histology and to differentiate benign from malignant types. MRI for scrotal pathology has proved to be a valuable second-level imaging modality that could help to elucidate diagnostic dilemmas found at US. Indeed, characterization of scrotal lesions at US may sometimes be difficult as a result of several limitations of this technique compared with MRI, which include the small field of view, operator dependence, and limited tissue characterization^23^. In selected cases MR could represent a useful adjunct for patients with inconclusive clinical and US findings, since it could modify and direct treatment strategies towards more conservative approaches, including biopsy, tumor enucleation and testicular-sparing surgery, or even clinical and imaging follow-up when deemed possible^6,24^. Nevertheless, a confident characterization of the nature of scrotal masses is not always achievable even with MRI. Not surprisingly, Radiomics represents a rapidly-growing translational field of research that has been applied to cancer care in an effort to find imaging biomarkers as decision support tools for clinical practice, given the increased number and availability of imaging data in oncology. Lung, breast, colorectal, renal cell, pancreatic, brain cancer and sarcoma have all been previously investigated through medical image processing and analysis^25,26^, whereas only one study^27^ has applied radiomic analysis to retroperitoneal nodal masses from germ cell testis cancer after chemotherapy.

In the literature, a previous study has tested the ability to discriminate between seminomas and non-seminomas through qualitative observation by the radiologist examining on MRI images morphologic features, including tumor volume, infiltrative margins, fibrovascular septa, necrosis^7^. This study reported high inter-radiologist agreement and accuracy of MRI findings in predicting histologic diagnosis of 91%. However, the number of patients was limited to 21 cases and the interpretation of MRI findings will always be dependent on radiologist expertise. Other studies have focused on quantitative MRI imaging, such as DWI with ADC values giving promising results in discriminating SGCT-NSGCT status with an AUC of 0.906^9,10,28,29^. The robustness of these results was also proven against different ROI definitions^28^. Furthermore, dynamic contrast enhanced (DCE)—MRI has been also proven to be a valuable semi-quantitative method to discriminate TGCT and TNGCT lesions with a maximum AUC of 0.89; however, these methods do not provide numerical data for a standardized assessment^10^. Recently, Zhang et al.^14^ proposed a radiomic signature based on multiple features able to discriminate quantitatively SGCT-NSGCT status with high reproducibility scoring an AUC of 0.979. Unfortunately, a radiomic signature comparison is hard to assess due to the complexity and high number of features employed for its development and it is beyond the scope of this study. We provide in this work the *Radiomic Quality Score* as Supplementary Material S3. Here we propose two classifiers to discriminate TGCT-TNGCT and SGCT-NSGCT status based on a selection of features. fivefold cross validation was employed to avoid overfitting and increase the robustness of the model; unfortunately, an internal validation represents a limitation, as an external dataset will be required to confirm the results. However, the data supporting the conclusions of this manuscript are available under request.

The two model shares VD as the strongest predictors whereas the other features are different. VD is the strongest predictor for both the models and is a shape feature which depicts the ratio of the volume of the lesion in comparison to a standard reference volume which in this case is the box containing the lesion itself. This feature can be considered MRI independent and this would be a strong benefit for the extension of the model to external datasets. The TGCT-TNGCT classifier is composed by two other features VFDI and SZLGLE that are an intensity-volume histogram and gray level zone size-based features. The SGCT-NSGCT classifier is based on other three features which are AD, quartile coefficient of dispersion and energy. AD is very similar in concept to VD however the two are not correlated and both contribute to the classification. Quartile coefficient of dispersion and energy are both histogram intensity-based features. It is interesting to notice how in general models rely on shape and first order histogram-based features. The only exception that we point out is the contribution of SZLGLE. This feature emphasises zone counts where small zone sizes and low grey levels are located however it is difficult to speculate on the lesion heterogeneity as the differences between TGCT and TNGCT are not so evident as can be noted in Fig. 3. Both the models present the limitation of class imbalance being TNGCT and NSGCT the minority classes. With regard to this limitation, the calculation of precision, recall and F1-score (which combines the two previous metrics) was performed for these models. The result obtained are encouraging. In particular, we obtained a 0.78 F1-score for NTGCT and 0.85 for NSGCT that are consider a fair level for model reliability.

Following these promising results, we believe that Radiomics can be integrated with other quantitative techniques such as ADC and DCE to improve testicular mass classification accuracy. We are aware that another limitation of this study lies in its retrospective nature and in the relatively low number of patients. Furthermore, the dependency of the features identified on contouring method and scanner vendors was not explored in this study.

In conclusion, our preliminary study shows that the radiomic measures obtained by scrotal MR image analysis may be useful in the diagnostic workup of testicular lesions, since they could add valuable information and help to discriminate among testicular neoplasms by differentiating germ cell from non-gem cell tumors, and SGCT from other histologies. However, our conclusion currently should be considered as a proof of concept because further independent validation with an external cohort of patients is required to assess whether other quantitative imaging features may improve the characterization of testicular lesions.

## Supplementary Information

Supplementary Material S1.

Supplementary Material S2.

Supplementary Material S3.

## Data Availability

The datasets considered in this work is available from the corresponding author on reasonable request.

## Abbreviations

AJCC: American Joint Committee on Cancer
ADC: Apparent diffusion coefficient
AD: Area density
AUC: Area under the curve
DWI: Diffusion-weighted imaging
DCE: Dynamic contrast-enanced imaging
IBSI: Image biomarker standardisation initiative
MRI: Magnetic resonance imaging
NSGCT: Nonseminomatous germ cell tumor
ROI: Region of interest
SGCT: Seminoma germ cell tumors
SERA: Standardized environment for radiomics analysis
SZLGLE: Small zone low grey level emphasis
SVM: Support vector machine
TGCT: Testicular germ cell tumor
TNGCT: Testicular non germ cell tumor
TPR: True positive rate
US: Ultrasonography
VD: Volume density
VFDI: Volume fraction difference between 10 and 90% Intensity
VOI: Volume of interest
