# Supplementary material for: The potential role of MR based radiomic biomarkers in the characterization of focal testicular lesions
Source: Sci Rep. 2021 Feb 10;11:3456. doi: 10.1038/s41598-021-83023-4 (PMC7875983; doi:10.1038/s41598-021-83023-4)
Supplement: Supplementary file 1 — Supplementary Material S1. [file 41598_2021_83023_MOESM1_ESM.docx]

**Title:** The potential role of MR based radiomic biomarkers in the characterization of focal testicular lesions.

**Authors:**  Giacomo Feliciani, Lorenzo Mellini, Aldo Carnevale, Anna Sarnelli, Enrico Menghi, Filippo Piccinini, Emanuela Scarpi, Emiliano Loi, Roberto Galeotti, Melchiore Giganti, Gian Carlo Parenti

**Supplemental Table 1**: **Patient demographics and visual features of the lesions.** The presence of a certain characteristic in the lesions is labelled by 1**.** HOMO – refers to the signal homogeneity, LOW SI is the relative intensity of the lesion compared to normal testicular parenchyma on T2w sequences, NECRO/HEMO is the presence of necrotic or hemorrhagic areas, CAPSULE refers to the presence of capsule. SEPTA and CE refer to the presence and contrast uptake of bandwise regions in T2w sequences.

| **ID** | **AGE** | **STAGE** | **HOMO** | **LOW SI** | **NECRO/HEMO** | **CAPSULE** | **SEPTA** | **CE** |
| --- | --- | --- | --- | --- | --- | --- | --- | --- |
| **NON GERMINOMAS** | | | | | | | | |
| T010 | 51 | nd | 0 | 0 | 0 | 0 | 0 | 0 |
| T016 | 33 | nd | 0 | 0 | 0 | 0 | 0 | 0 |
| T018 | 22 | nd | 0 | 1 | 1 | 1 | 0 | 0 |
| T019 | 30 | nd | 0 | 1 | 0 | 1 | 0 | 0 |
| T020 | 40 | nd | 1 | 0 | 0 | 1 | 0 | 0 |
| T022 | 63 | nd | 0 | 1 | 0 | 1 | 0 | 0 |
| T023 | 67 | nd | 1 | 1 | 0 | 0 | 0 | 0 |
| T027 | 35 | nd | 0 | 1 | 0 | 1 | 0 | 0 |
| T030 | 46 | nd | 1 | 1 | 0 | 0 | 0 | 0 |
| T039 | 7 | nd | 1 | 1 | 0 | 0 | 0 | 0 |
| T041 | 58 | nd | 0 | 1 | 0 | 1 | 0 | 0 |
| **Ratio 1 vs Total (%)** | | | 27 | 72 | 9 | 58 | 54 | 0 |
| **GERMINOMAS - NON SEMINOMAS** | | | | | | | | |
| T017 | 37 | pT1a | 0 | 1 | 0 | 0 | 1 | 1 |
| T024 | 26 | pT2 | 0 | 0 | 1 | 0 | 0 | 0 |
| T025 | 24 | pT2 | 0 | 1 | 1 | 0 | 0 | 0 |
| T028 | 41 | pT3 | 0 | 1 | 1 | 0 | 0 | 0 |
| T031 | 32 | pT1 | 0 | 1 | 1 | 1 | 0 | 0 |
| T036 | 26 | pT1 | 0 | 0 | 1 | 0 | 0 | 0 |
| T038 | 26 | pT2 | 0 | 0 | 1 | 0 | 0 | 0 |
| T040 | 32 | pT2 | 0 | 1 | 1 | 1 | 0 | 0 |
| T048 | 46 | pT2 | 0 | 0 | 1 | 0 | 0 | 0 |
| **Ratio 1 vs Total (%)** | | | 0 | 55 | 89 | 22 | 11 | 11 |
| **SEMINOMAS** | | | | | | | | |
| T001 | 37 | pT2 | 0 | 1 | 1 | 0 | 1 | 1 |
| T002 | 43 | pT1b | 0 | 1 | 1 | 0 | 1 | 1 |
| T004 | 54 | pT2 | 0 | 1 | 1 | 0 | 1 | 1 |
| T005x | 38 | pT1b | 0 | 1 | 0 | 0 | 1 | 1 |
| T005y | 38 | pT1a | 1 | 1 | 1 | 1 | 0 | 0 |
| T006 | 31 | pT1a | 0 | 1 | 0 | 0 | 0 | 0 |
| T007 | 36 | pT1a | 1 | 1 | 1 | 0 | 1 | 1 |
| T008 | 37 | pT2 | 1 | 1 | 0 | 0 | 0 | 0 |
| T009 | 39 | pT1b | 1 | 1 | 0 | 0 | 1 | 1 |
| T011 | 60 | pT3 | 0 | 1 | 0 | 0 | 1 | 1 |
| T012 | 26 | pT1a | 0 | 1 | 0 | 0 | 1 | 1 |
| T013 | 36 | pT1a | 1 | 1 | 0 | 1 | 0 | 0 |
| T015 | 50 | pT2 | 0 | 1 | 1 | 0 | 1 | 1 |
| T021 | 44 | pT2 | 0 | 0 | 1 | 0 | 1 | 1 |
| T029 | 31 | pT1a | 1 | 1 | 0 | 0 | 1 | 1 |
| T032 | 30 | pT1a | 0 | 1 | 0 | 0 | 1 | 1 |
| T033 | 35 | pT1a | 0 | 1 | 0 | 0 | 0 | 0 |
| T034 | 44 | pT2 | 0 | 1 | 1 | 0 | 1 | 1 |
| T035 | 23 | pT1b | 1 | 1 | 0 | 0 | 1 | 1 |
| T037 | 40 | pT2 | 0 | 1 | 0 | 0 | 0 | 0 |
| T042 | 35 | pT1b | 0 | 1 | 0 | 0 | 1 | 1 |
| T044 | 12 | T1 | 0 | 0 | 0 | 1 | 0 | 0 |
| T045 | 49 | pT1a | 0 | 1 | 1 | 1 | 1 | 0 |
| T047 | 43 | pT2 | 0 | 1 | 0 | 0 | 0 | 0 |
| **Ratio 1 vs Total (%)** | | | 29 | 92 | 37 | 17 | 67 | 63 |
