# Supplementary material for: The potential role of MR based radiomic biomarkers in the characterization of focal testicular lesions
Source: Sci Rep. 2021 Feb 10;11:3456. doi: 10.1038/s41598-021-83023-4 (PMC7875983; doi:10.1038/s41598-021-83023-4)
Supplement: Supplementary file 2 — Supplementary Material S2. [file 41598_2021_83023_MOESM2_ESM.docx]

**Radiomics Quality Score: according to** (1)

| **Criteria** | | **Points** |
| --- | --- | --- |
| 1 | Image protocol quality - well-documented image protocols (for example, contrast, slice thickness, energy, etc.) and/or usage of public image protocols allow reproducibility/replicability | + 1 (if protocols are well-documented) |
| 2 | Multiple segmentations - possible actions are: segmentation by different physicians/algorithms/software, perturbing segmentations by (random) noise, segmentation at different breathing cycles. Analyse feature robustness to segmentation variabilities | + 0 Only one physician segmented lesions |
| 3 | Phantom study on all scanners - detect inter-scanner differences and vendor-dependent features. Analyse feature robustness to these sources of variability | + 0 no phantom was employed in the study |
| 4 | Imaging at multiple time points - collect images of individuals at additional time points. Analyse feature robustness to temporal variabilities (for example, organ movement, organ expansion/ shrinkage) | + 0 not possible with retrospective study |
| 5 | Feature reduction or adjustment for multiple testing - decreases the risk of overfitting. Overfitting is inevitable if the number of features exceeds the number of samples. Consider feature robustness when selecting features | + 3 we used non parametric tests plus 5-fold cross validation to avoid overfitting |
| 6 | Multivariable analysis with non radiomics features (for example, EGFR mutation) - is expected to provide a more holistic model. Permits correlating/inferencing between radiomics and non radiomics features | + 1 logistic regressions are showed in the main document. |
| 7 | Detect and discuss biological correlates - demonstration of phenotypic differences (possibly associated with underlying gene–protein expression patterns) deepens understanding of radiomics and biology | + 1 we furnish neoplasms visual characteristics |
| 8 | Cut-off analyses - determine risk groups by either the median, a previously published cut-off or report a continuous risk variable. Reduces the risk of reporting overly optimistic results | N/A – small cohort |
| 9 | Discrimination statistics - report discrimination statistics (for example, C‑statistic, ROC curve, AUC) and their statistical significance (for example, p‑values, confidence intervals). One can also apply resampling method (for example, bootstrapping, cross-validation) | + 1 (ROC and confusion matrices are shown in text) + 1 (Cross validation) |
| 10 | Calibration statistics - report calibration statistics (for example, Calibration-in‑the-large/slope, calibration plots) and their statistical significance (for example, *P*‑values, confidence intervals). One can also apply resampling method (for example, bootstrapping, cross-validation) | 0 |
| 11 | Prospective study registered in a trial database - provides the highest level of evidence supporting the clinical validity and usefulness of the radiomics biomarker | 0 |
| 12 | Validation - the validation is performed without retraining and without adaptation of the cut-off value, provides crucial information with regard to credible clinical performance | - 5 |
| 13 | Comparison to ‘gold standard’ - assess the extent to which the model agrees with/is superior to the current ‘gold standard’ method (for example, TNM-staging for survival prediction). This comparison shows the added value of radiomics | + 2 comparison with previous study are discussed in discussion session |
| 14 | Potential clinical utility - report on the current and potential application of the model in a clinical setting (for example, decision curve analysis). | + 2 |
| 15 | Cost-effectiveness analysis - report on the cost-effectiveness of the clinical application (for example, QALYs generated) | + 1 |
| 16 | Open science and data - make code and data publicly available. Open science facilitates knowledge transfer and reproducibility of the study | + 1 (if scans are open source) + 1 (if region of interest segmentations are open source) + 1 (if code is open source) + 1 (if radiomics features are calculated on a set of representative ROIs and the calculated features and representative ROIs are open source) |
| Total points ( 11 ) | | |

1. Lambin P, Leijenaar RTH, Deist TM, et al.: Radiomics: the bridge between medical imaging and personalized medicine. *Nat Rev Clin Oncol* 2017; 14:749.
