# Supplementary material for: The potential role of MR based radiomic biomarkers in the characterization of focal testicular lesions
Source: Sci Rep. 2021 Feb 10;11:3456. doi: 10.1038/s41598-021-83023-4 (PMC7875983; doi:10.1038/s41598-021-83023-4)
Supplement: Supplementary file 3 — Supplementary Material S3. [file 41598_2021_83023_MOESM3_ESM.docx]

**Features mathematical formulas and descriptions**

**Volume density (axis-aligned bounding box)**

Volume density is the fraction of the ROI volume and a comparison volume. Here the comparison volume is that of the axis-aligned bounding box (AABB) of the ROI mesh vertex set X_vx_ or the ROI mesh convex hull vertex set X_vx;convex_. Both vertex sets generate an identical bounding box, which is the smallest box enclosing the vertex set, and aligned with the axes of the reference frame.

$${VD}_{morph}=\frac{V}{V_{aabb}}$$

### Where V is the ROI volume and $\boldsymbol{V}_{\boldsymbol{aabb}}$ is the volume of the axis-aligned bounding box (AABB)

**Area density (axis-aligned bounding box)**

Conceptually similar to the volume density (AABB) feature, area density considers the ratio of the ROI surface area and the surface area Aaabb of the axis-aligned bounding box enclosing the ROI mesh vertex set X_vx_. The bounding box is identical to the one used for computing the volume density (AABB) feature. Thus:

$${AD}_{morph}=\frac{A}{A_{aabb}}$$

### Volume at intensity fraction and Volume fraction difference between intensity fractions

The volume at intensity fraction Vx is the largest volume fraction _ that has an intensity fraction of at least x%. This differs from conceptually similar dose-volume histograms used in radiotherapy planning, where V_10_ would indicate the volume fraction receiving at least 10 Gy planned dose. El Naqa et al. (1) defined both V_10_ and V_90_ as features. Volume fraction difference between intensity fractions is the difference between the volume fractions at two different intensity fractions, e.g.V_10_ - V_90._

### Gray Level Size Zone Matrix (GLSZM) Features

A Gray Level Size Zone (GLSZM) quantifies the gray level zones in an image. A gray level zone is defined as the number of connected voxels that share the same gray level intensity. A voxel is considered connected if the distance is 1 according to the infinity norm (26-connected region in a 3D, 8-connected region in 2D).

In a gray level size zone matrix *P(i,j)*, the *(i,j)*^th^ element equals to the number of zones with gray level *i* and size *j* appear in image. Contrary to GLCM and GLRLM, the GLSZM is rotation independent, with only one matrix calculated for all directions in the ROI.

Let:

- *N_g_* be the number of discrete intensity values in the image
- *N_z_* be the number of discrete zone sizes in the image
- *N_v_* be the number of voxels in the image
- *N_s_* be the number of zones in the ROI, which is equal to
  $\sum_{i=1}^{Ng} \sum_{j=1}^{Ns} s\left( i,j \right)$ and $1\leq N_{z}\leq N_{g}$
- *S(i,j)* be the size zone matrix
- *s(i,j)* be the normalized size zone matrix, defined as $s\left( i,j \right)=\frac{S\left( i,j \right)}{N_{z}}$

### small zone low grey level emphasis (SZLGLE)

This feature emphasises zone counts within the upper left quadrant of the GLSZM, where small zone sizes and low grey levels are located. It is defined as:

$$F_{szm.szgle}=\frac{1}{N_{s}}\sum_{i=1}^{N_{g}} \sum_{j=1}^{N_{z}} \frac{S(i,j)}{i^{2}j^{2}}$$

1. El Naqa I, Grigsby P, Apte A, Kidd E: Exploring feature-based approaches in PET images for predicting cancer treatment outcomes. *Pattern Recognit* 2009; 42:1162–1171.

### Intensity-based statistical features

The intensity-based statistical features describe how intensities within the region of interest (ROI) are distributed. The features in this set do not require discretisation, and may be used to describe a continuous intensity distribution. Intensity-based statistical features are not meaningful if the intensity scale is arbitrary.

The set of intensities of the Nv voxels included in the ROI intensity mask is denoted as:

$$X_{gl}= \left\{ X_{gl,1},X_{gl,2}....X_{gl,N} \right\}$$

### Intensity-based quartile coeffcient of dispersion

The quartile coefficient of dispersion is defined as:

$$F_{stat.qcod}=\frac{P_{75}-P_{25}}{P_{75}+P_{25}}$$

P_25_ and P_75_ are the 25th and 75th percentile of X_gl_, respectively.

**Intensity-based energy**

The energy of X_gl_ is defined as:

$$F_{stat.energy}=\sum_{k=1}^{N_{v}} X_{gl,k}^{2}$$
